# Sex differences in the transcriptional response to acute inflammatory challenge: A randomized controlled trial of endotoxin

**DOI:** 10.1016/j.bbih.2024.100840

**Published:** 2024-08-02

**Authors:** Chloe C. Boyle, Steve W. Cole, Naomi I. Eisenberger, Richard Olmstead, Elizabeth C. Breen, Michael R. Irwin

**Affiliations:** a Norman Cousins Center for Psychoneuroimmunology, UCLA, USA; b Jane and Terry Semel Institute for Neuroscience and Human Behavior at UCLA, Department of Psychiatry and Biobehavioral Sciences, David Geffen School of Medicine, USA; c Division of Hematology-Oncology, Department of Medicine, UCLA School of Medicine, USA; d Department of Psychology, University of California, Los Angeles, CA, USA

**Keywords:** Sex, Inflammation, Transcription factors, Gene expression, Immune system

## Abstract

**Background:**

Sex differences in immune-based disorders are well-established, with female sex associated with a markedly heightened risk of autoimmune disease. Female sex is also overrepresented in other conditions associated with elevated inflammation, including depression, chronic pain, and chronic fatigue. The mechanisms underlying these disparities are unclear. This study used an experimental model of inflammatory challenge to interrogate molecular mechanisms that may contribute to female vulnerability to disorders with an inflammatory basis.

**Method:**

In this analysis of a secondary outcome from a randomized controlled trial, 111 participants (67 female) received either a bolus injection of endotoxin (n = 59) or placebo (n = 52). Participants provided blood samples before and 0.5 h post-injection for assessment of differential activation of key pro-inflammatory (i.e., activator protein (AP)-1; nuclear factor (NF)-κB) and immunoregulatory (i.e., glucocorticoid receptor (GR); cAMP response element binding protein (CREB)) signaling pathways via genome-wide expression profiling and promoter-based bioinformatics analyses.

**Results:**

Relative to males, females exhibited greater endotoxin-induced increases in bioinformatic measures of CREB transcription factor activity (*p's* < 0.01). However, contrary to hypotheses, female vs. male sex was not associated with greater increases in activation of NF-κB, AP-1, or GR in response to endotoxin vs. placebo administration.

**Conclusions:**

This work suggests CREB signaling as a critical upstream biological pathway that should be further interrogated as a mechanism of female vulnerability to immune-related disorders. Future work should clarify whether increased CREB signaling indicates sex differences in activity of the sympathetic nervous system or other physiological pathways that signal through CREB, such as prostaglandin release.

## Highlights

• Randomized controlled trial of acute inflammatory challenge found that female (vs male) participants exhibited greater endotoxin-induced increases in bioinformatic measures of CREB transcription factor activity.
• There were no sex differences in endotoxin vs. placebo induced increases in activation of NF-κB, AP-1, or GR transcription factor activity.
• This work implicates CREB signaling as a critical upstream biological mechanism of female vulnerability to immune-related disorders such as depression.

## 1 Introduction

Sex-based disparities in immune-related health outcomes are well-documented. Female sex is associated with a markedly heightened risk of autoimmune disease, with females comprising over 80% of affected individuals for several autoimmune disorders (Libert et al., 2010). Moreover, female sex is overrepresented in multiple conditions associated with elevated markers of inflammation, including depression (Kropp and Hodes, 2023), chronic pain (Irwin et al., 2023), and chronic fatigue (Yoon et al., 2023; Strawbridge et al., 2019). Beyond these disparities, females also show a stronger innate and humoral immune responses to infection and vaccination (Fish, 2008; Klein et al., 2010), higher systemic levels of inflammatory biomarkers such as C-reactive protein (Woloshin and Schwartz, 2005), and greater cellular immune activation following sleep deprivation (Irwin et al., 2010), as compared to males. The mechanisms underlying these differences remain unclear. The purpose of the current study was to interrogate molecular mechanisms that may contribute to female vulnerability to disorders with an inflammatory basis.

Experimental models of systemic inflammation enable a precise interrogation of the immune system. Among these models, the endotoxin challenge, which involves the controlled administration of a component of the outer membrane of Gram-negative bacteria, stands out for its ability to mimic a naturally occurring inflammatory immune response induced by pathogenic bacteria (Lasselin et al., 2021). Endotoxin administration triggers an inflammatory cascade and elicits increases in proinflammatory cytokines comparable to those found in inflammatory disorders and chronic infections, with effects limited to hours (Andreasen et al., 2008). Some studies have shown more robust increases in endotoxin-induced pro-inflammatory cytokines in females versus males (Engler et al., 2016) whereas others have shown no sex differences (Moieni et al., 2015a). Moreover, while research findings on sex differences in associated behavioral symptoms, including depressive mood, fatigue, and pain, are inconsistent (Lasselin et al., 2018), the few instances where differences are identified typically show that females exhibit greater vulnerability than males (Moieni et al., 2015a; Lasselin et al., 2018; Eisenberger et al., 2009; Karshikoff et al., 2015; Benson et al., 2017). However, very few studies have evaluated the upstream molecular mechanisms that may contribute to potential sex differences in vulnerability to the effects of inflammatory challenge, or the well-documented female preponderance of autoimmune disorders, depression, and chronic pain and fatigue. Those studies that have examined changes in gene expression and transcription factor activity following endotoxin challenge have reported on sample sizes too small to evaluate sex differences (Calvano et al., 2005; Talwar et al., 2006).

Thus, the current study (an analysis of a secondary outcome of a completed randomized controlled trial) tested the hypothesis that an acute inflammatory challenge would be associated with differential activation of inflammatory and immunoregulatory signaling pathways in females as compared to males in a large sample of 111 adults. We focused on two key pro-inflammatory transcription factors that are activated by endotoxin administration and subsequent toll-like receptor 4 signaling, nuclear factor (NF)-κB and activator protein (AP)-1 (Chen et al., 2017), as well as two immunomodulatory neuroendocrine mediators, the glucocorticoid receptor (GR; indicative of hypothalamic-pituitary-adrenal (HPA) axis activity) and the cAMP response element binding protein (CREB; indicative of sympathetic nervous system (SNS) activity). Both GR (Salkowski and Vogel, 1992) and CREB (Ciesielska et al., 2021) have been shown to be upregulated in response to acute inflammatory challenge. We hypothesized that female vs. male sex would be associated with increased activation of NF-κB, AP-1, GR, and CREB in response to endotoxin vs. placebo administration.

## 2 Methods

### 2.1 Participants and procedures

One hundred and fifteen healthy participants (69 female; mean age 24.2 ± 6.6 years) completed a randomized study of endotoxin vs. placebo administration. Inclusion and exclusion criteria were previously described (Moieni et al., 2015a, 2015b). Briefly, participants were free from any inflammatory disease or concurrent infection and did not use anti-inflammatory medications. Two female and two male participants did not provide blood samples, so the final analytical sample included 111 participants (mean age 24.3 ± 6.7 years; 67 female); there were no sex differences in age, body mass index (BMI) or race/ethnicity (all *p*'s > 0.06). Fifty-nine participants received endotoxin (37 female) and 52 received placebo (30 female) (Table 1). All subjects provided written consent; procedures were approved by the UCLA Human Subjects Protection Committee.

**Table 1:** Sample Characteristics of All Participants, Stratified by Endotoxin vs Placebo Exposure and Sex.

	Total (*n* = 111)	All Endotoxin (n = 59)		All Placebo (n = 52)		Tests for group X sex differences^a^
		Female (n = 37)	Male (*n* = 22)	Female (*n* = 30)	Male (*n* = 22)	
Age, years mean (range)	24.3 (18–50)	24.9 (18–50)	25.5 (19–45)	21.4 (18–29)	26.0 (18–47)	*p* = 0.11
BMI, kg/m^2^ mean (range)	23.8 (18.5–29.9)	23.9 (18.5–29.9)	24.4 (20.4–27.8)	23.2 (19–29.9)	23.8 (18.9–28.6)	*p* = 0.92
Race/ethnicity *n* (%)						*p* = 0.15
Asian	33 (30%)	9 (24%)	9 (41%)	7 (23%)	8 (36%)	
Black/Other	13 (12%)	5 (14%)	2 (9%)	5 (17%)	1 (5%)	
Latinx	23 (20%)	10 (27%)	5 (23%)	8 (27%)	0 (0%)	
White	42 (38%)	13 (35%)	6 (27%)	10 (33%)	13 (59%)	

Abbreviations: BMI = body mass index. a: Age and BMI tested by multiple regression; Race/ethnicity tested by chi square.

The study was conducted between March 2011 and August 2013 (NCT01671150) using a randomized, double-blind, placebo-controlled design. Results previously reported include among others varying depressed mood responses due to sex (Moieni et al., 2015a), varying transcriptional response due to psychosocial factors (Moieni et al., 2015b; Irwin et al., 2018), and baseline transcriptome profiles as predictors of depressive mood (Cho et al., 2019). No prior report has tested for sex differences in the genome-wide transcriptional response to endotoxin challenge.

Each participant was randomly assigned to receive either low-dose endotoxin (0.8 ng/kg of body weight, E. coli group O:113 BB-IND 12948) or placebo (same volume of 0.9% saline) as an intravenous bolus by a computer-generated 1:1 allocation algorithm. Low-dose endotoxin administration mimics the increases in inflammation that are found in inflammatory disorders and infections (Breen et al., 1990; Ishihara and Hirano, 2002; Mangge et al., 1995). Blood was collected for gene expression analyses at two timepoints (T0, baseline prior to endotoxin or placebo injection, and T0.5, 30 min after injection) because peak RNA response precedes peak response in proteins (e.g., IL-6, TNF-α), which occurs at approximately 2 and 1 h post-injection, respectively (Moieni et al., 2015b).

### 2.2 Assessment of transcription factor activity

Change in pro-inflammatory and immunoregulatory signaling in peripheral blood mononuclear cells (PBMC) was assessed using bioinformatic quantification of activity in 4 a-priori-selected transcription control pathways (NF-κB, AP-1, GR, and CREB). These bioinformatic inferences were derived from linear model analysis of (log2-transformed) gene expression profiling data. Paired blood samples for gene expression were collected via an intravenous catheter with heparin lock in Cell Preparation Tubes (CPT, Becton-Dickinson) at T0 and T0.5 and held at room temperature until both samples had been obtained. PBMC were harvested following centrifugation per the manufacturer's protocol, pelleted (approximately 2-5 x 10^6^ PBMC/pellet), and then resuspended in RLT lysis Buffer (Qiagen) with β−2-mercaptoethanol and frozen immediately at −80 °C until subsequent RNA extraction (Qiagen RNeasy) using an automated nucleic acid processing system (Qiagen QIAcube) (Moieni et al., 2015a; Irwin et al., 2018). RNA samples were tested for suitable quantity (RiboGreen RNA) and quality (RNA integrity assessment by Agilent TapeStation).

Genome-wide transcriptional profiling was conducted on isolated PBMC RNA using Ambion TotalPrep cRNA targets hybridized to Illumina HT-12 v4 bead arrays, as performed in the UCLA Neuroscience Genomics Core Laboratory following the manufacturer's standard protocol (Moieni et al., 2015a; Irwin et al., 2018). All paired samples (T0 and T0.5 from the same participant) yielded sufficient RNA for analysis, were assayed in a single batch, and provided valid results according to standard data quality metrics (e.g., median probe fluorescence intensity >100 units).

### 2.3 Analytic approach

Analyses were conducted using SAS version 9.4 and Stata 16.1. The aim was to evaluate whether participant sex significantly modified the effect of acute inflammatory challenge on bioinformatically inferred activity of cardinal pro-inflammatory transcription factors (NF-κB, AP-1) and key neuroendocrine transcription factors that regulate inflammation (glucocorticoid receptor/GR for HPA and CREB for the SNS). Towards this end, transcriptome-wide analyses took as input all gene transcripts showing >1.2-fold change (Irwin et al., 2018; Miller et al., 2009) in expression in association with a condition (endotoxin vs. placebo) by time (T0 vs. T0.5) by sex (female vs. male) interaction using the TELiS promoter-based bioinformatics system (Cole et al., 2005).

Transcription factor activity was assessed using the following Transfac transcription-factor binding motif weight matrices for NF-κB (V$NFKB_Q6), AP-1 (V$AP1_Q6), CREB (V$CREB_Q4), and GR (V$GR_Q6). The log-ratio of transcription factor-binding motifs (TFBM) in the promoter sequences of up vs. down-regulated genes was assessed with results averaged over 9 parametric combinations of 3 promoter sequences lengths (300, 600, 1200) and 3 stringencies for motif detection (Transfac mat_sim values ≥ 0.80, 0.90, and 0.95) and standard errors derived by bootstrapping.

## 3 Results

### 3.1 Effect of acute inflammatory challenge on gene expression and transcription factor activity as a Function of sex

To address this study's primary hypothesis, we identified all genes that demonstrated >1.2 fold differential change in average expression in relation to the endotoxin by time by sex interaction. Relatively few genes met that threshold (47 up-regulated, 8 down-regulated), but those that were more strongly upregulated by endotoxin in females included pro-inflammatory mediators (*TNF, CXCL8*/IL8, *FOS, JUN, CCL3*) as well as *ADRB2* – the beta2-adrenergic receptor.

**Fig. 1:**
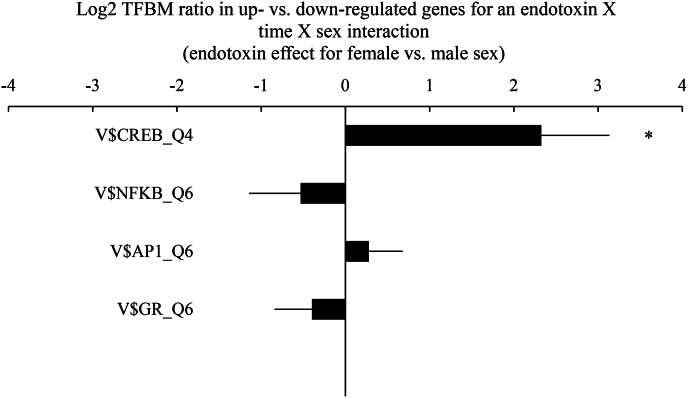
Transcription factor-binding motif (TFBM) ratio for transcription factors, the cAMP response element binding protein (CREB), nuclear factor (NF)-κB, activator protein (AP) −1, and glucocorticoid receptor (GR), as measured by promoter-based bioinformatic analyses of all genes that showed >1.2 fold change over time in association with the condition by time by sex interaction. In the endotoxin vs. placebo group, female sex was associated with greater pre to post-injection increases in CREB activity (p = 0.004), but no differential change in NF-κB, AP-1, or GR activity (all *p*'s > 0.3). Data represent log2 ratios (null value = 1.0 fold = 0 log2).

To assess the role of the targeted transcription control pathways in these empirical transcriptome differences, genes were subject to TELiS promoter-based bioinformatics analysis (see Fig. 1 for an overview of findings). Contrary to hypotheses, there was no evidence for sex differences in the degree of endotoxin-induced change in pro-inflammatory signaling for the NF-κB (*p* = 0.39) or AP-1 (*p* = 0.48) transcription factor. In terms of potential immunomodulatory neuroendocrine mediators, there was no change in the anti-inflammatory GR transcription factor (*p* = 0.37), but there was evidence for differential CREB transcription factor activity (indicative of SNS activity). Specifically, endotoxin increased bioinformatic measures of CREB TFBM activity over time to a greater degree in female as compared to male participants in the endotoxin vs. placebo condition from baseline to 0.5 h post-injection (5.01-fold ratio of binding sites in up-vs down-regulated genes; log_2_ ratio = 2.33 ± 0.81 SE; *p* = 0.004).

## 4 Discussion

The purpose of the current study was to interrogate molecular mechanisms that may contribute to sex-based disparities in disorders with an inflammatory basis. Using a robust and reliable experimental model of acute inflammatory challenge, along with a sample size sufficient to evaluate sex differences, this work identifies CREB signaling as a critical upstream biological pathway that should be further interrogated as a mechanism of female vulnerability to immune-related disorders. By contrast, there was no evidence for sex differences in endotoxin-induced activity of the canonical pro-inflammatory transcription factors NF-κB or AP-1, despite increased expression of multiple pro-inflammatory genes. Thus, the sex differences in gene expression do not appear to be due to differential activation of classical cytokine-induced pro-inflammatory signaling pathways. There was also no evidence for differential activation of GR, which might have suggested an anti-inflammatory pathway as a plausible mechanism.

Increased CREB signaling could indicate potential differences in activity of the SNS, which activates CREB via beta-adrenergic receptors. Alternatively, increased CREB activity could indicate increased activity of another physiological pathway that signals through CREB, such as prostaglandins (Saper et al., 2012). Both proinflammatory cytokines and prostaglandins are implicated in activating sickness behavior (Saper et al., 2012), and COX inhibitors (which convert arachidonic acid to prostaglandin H2) can reduce sickness symptoms such as pain, fever, and loss of appetite. Moreover, preclinical and clinical studies have shown sex differences in prostaglandin signaling, with greater neuronal expression of the enzyme prostaglandin D2 synthase in females (Shen et al., 2023). Thus, these results may indicate sex differences in the inflammatory response to endotoxin are operating through heightened prostaglandin production in females vs. males, as opposed to differences in the release of pro-inflammatory cytokines. Indeed, our previous report of this sample found no sex differences in the release of IL-6 or TNF-α following endotoxin vs. placebo challenge (Moieni et al., 2015a).

Prior studies evaluating gene expression in response to an endotoxin challenge have found a similarly small number of differentially expressed genes (Talwar et al., 2006; Prabhakar et al., 2005), and to our knowledge none have conducted bioinformatics analyses to characterize upstream mediators of the systemic inflammatory response, or tested for sex differences in this response. Indeed, many endotoxin studies were typically only conducted with male participants, with the first study on behavioral effects that included females published in 2009 (Eisenberger et al., 2009). Thus, this work represents a notable contribution to the literature. Limitations include an absence of data on menstrual cycle phase or use of hormonal contraception, both of which could modulate the inflammatory response given that sex hormones play a role in immune regulation (Fish, 2008). However, hormone differences cannot fully explain sex differences in immune responses or disorders as these differences can be evident across the lifespan (Lasselin et al., 2018; Casimir et al., 2013). Like many endotoxin studies, this sample consisted primarily of younger adults, so results are specific to that age group. Moreover, there are no direct measures of downstream protein production (or CREB transcription factor activation) available to validate the RNA-based findings identified in this report. Confirmation of these bioinformatic inferences represents an important topic for future research. Despite these limitations, this work underscores the importance of integrating sex as a biological variable in research on immune-related disorders, revealing that differences in the response to an inflammatory challenge are not uniform across sex and may involve distinct molecular pathways such as CREB signaling and, potentially, prostaglandin production and/or beta-adrenergic signaling. Future research should expand on these findings by exploring the role of sex hormones and other physiological mediators across diverse age groups, to better understand the complex interplay between sex, inflammation, and immune regulation. This will pave the way for more personalized approaches to the treatment and management of inflammatory disorders, taking into account sex-specific vulnerabilities.

## Funding and disclosure

This research was funded by an R01 from 10.13039/100000025NIMH to NIE (5R01MH091352). This research was additionally supported by the following from NIH to Dr. Irwin (R01AG034588; R01AG026364; R01CA160245-01; R01CA119159; R01HL095799; R01DA032922) and by NIH/National Center for Advancing Translational Science (10.13039/100006108NCATS) 10.13039/100016206UCLA CTSI Grant Number UL1TR001881. 10.13039/100026020CCB was supported by the 10.13039/100000002National Institutes of Health, 10.13039/100000049National Institute on Aging (1K01AG072049-01A1).

## CRediT authorship contribution statement

**Chloe C. Boyle:** Conceptualization, Data curation, Visualization, Writing – original draft, Writing – review & editing. **Steve W. Cole:** Conceptualization, Formal analysis, Methodology, Resources, Writing – review & editing. **Naomi I. Eisenberger:** Conceptualization, Data curation, Funding acquisition, Investigation, Methodology, Resources, Writing – review & editing. **Richard Olmstead:** Conceptualization, Data curation, Writing – review & editing. **Elizabeth C. Breen:** Data curation, Investigation, Methodology, Writing – review & editing. **Michael R. Irwin:** Conceptualization, Funding acquisition, Investigation, Methodology, Project administration, Resources, Supervision, Writing – review & editing.

## Declaration of competing interest

The authors declare that they have no known competing financial interests or personal relationships that could have appeared to influence the work reported in this paper.

## Data Availability

Data will be made available on request.
